# Integration of thermal imaging and neural networks for mechanical strength analysis and fracture prediction in 3D-printed plastic parts

**DOI:** 10.1038/s41598-022-12503-y

**Published:** 2022-05-27

**Authors:** Daniil A. Boiko, Victoria A. Korabelnikova, Evgeniy G. Gordeev, Valentine P. Ananikov

**Affiliations:** Zelinsky Institute of Organic Chemistry, Russian Academy of Sciences, Leninsky Pr. 47, Moscow, 119991 Russia

**Keywords:** Design, synthesis and processing, Polymers

## Abstract

Additive manufacturing demonstrates tremendous progress and is expected to play an important role in the creation of construction materials and final products. Contactless (remote) mechanical testing of the materials and 3D printed parts is a critical limitation since the amount of collected data and corresponding structure/strength correlations need to be acquired. In this work, an efficient approach for coupling mechanical tests with thermographic analysis is described. Experiments were performed to find relationships between mechanical and thermographic data. Mechanical tests of 3D-printed samples were carried out on a universal testing machine, and the fixation of thermal changes during testing was performed with a thermal imaging camera. As a proof of concept for the use of machine learning as a method for data analysis, a neural network for fracture prediction was constructed. Analysis of the measured data led to the development of thermographic markers to enhance the thermal properties of the materials. A combination of artificial intelligence with contactless nondestructive thermal analysis opens new opportunities for the remote supervision of materials and constructions.

## Introduction

The increase in the strength of materials and constructions is one of the main tasks of modern industry and engineering^1^. There are two main factors that affect the properties of the resulting material: composition (including changes in the bulk material and combining multiple materials together) and structure. Additive manufacturing—a set of technologies of layer-by-layer 3D-object printing—affects both factors^2,3^, provides unique possibilities for printing complex objects^4^, and shows high sustainability^5^ and opening recycling opportunities^6,7^.

In the last decade, additive technologies have become widespread not only as prototyping methods but also as methods for final product manufacturing^8,9^. This is due to the dramatic reduction in the cost of 3D printers using the technologies of fused deposition modeling (FDM, also known as FFF), photopolymerization (SLA, DLP), and selective laser sintering of plastic powder (SLS), as well as the availability of computer-aided design systems^10,11^. The fundamental features of 3D printing are low cost for organizing a fully functional production pipeline, low operating costs for entry-level equipment, waste-free manufacturing processes, high efficiency in the use of construction materials, and a “manufacturing on-demand” business model^12,13^.

Currently, 3D printing technologies are most effective for the one-off and small-batch manufacturing of products with complex geometries. Therefore, 3D printing has found wide application in scientific laboratories to create customized components for scientific instruments and entire laboratory installations^14–16^, in mechanical engineering^17^, in medicine^18^ for the manufacturing of prostheses^19^, dental aligners^20^, personalized hearing aids^21^, and other medical devices. Today, there is no area of material production that does not use 3D printing to a greater or lesser extent.

Since 3D printing is a tool for the manufacturing of functional parts, the range of construction materials for additive manufacturing has expanded rapidly in recent years^22,23^. If until recently the main requirement for such materials was the convenience of printing and the ability to impart the necessary aesthetic properties to products, then now mechanical strength^24,25^, chemical stability^26^, thermal and electrical conductivity^27–29^, and tribotechnical characteristics^30^ are at the forefront. A distinctive feature of products obtained by 3D printing methods is a layered structure, especially pronounced in the case of FDM technology, which is currently the most widespread additive manufacturing technology. This leads to a significant anisotropy of the mechanical and thermophysical properties of FDM parts^31–36^. Therefore, the geometrical arrangement of the structural material within one layer and the orientation of the layers of the material relative to the direction of the mechanical load are important parameters for controlling the strength of the final products, along with the type of thermoplastic polymer^37^. In addition, the strength of FDM parts is significantly influenced by the geometry and degree of internal filling of the part, bed temperature, and printing temperature influencing the fusion of the polymer layers^38–40^.

**Figure 1:**
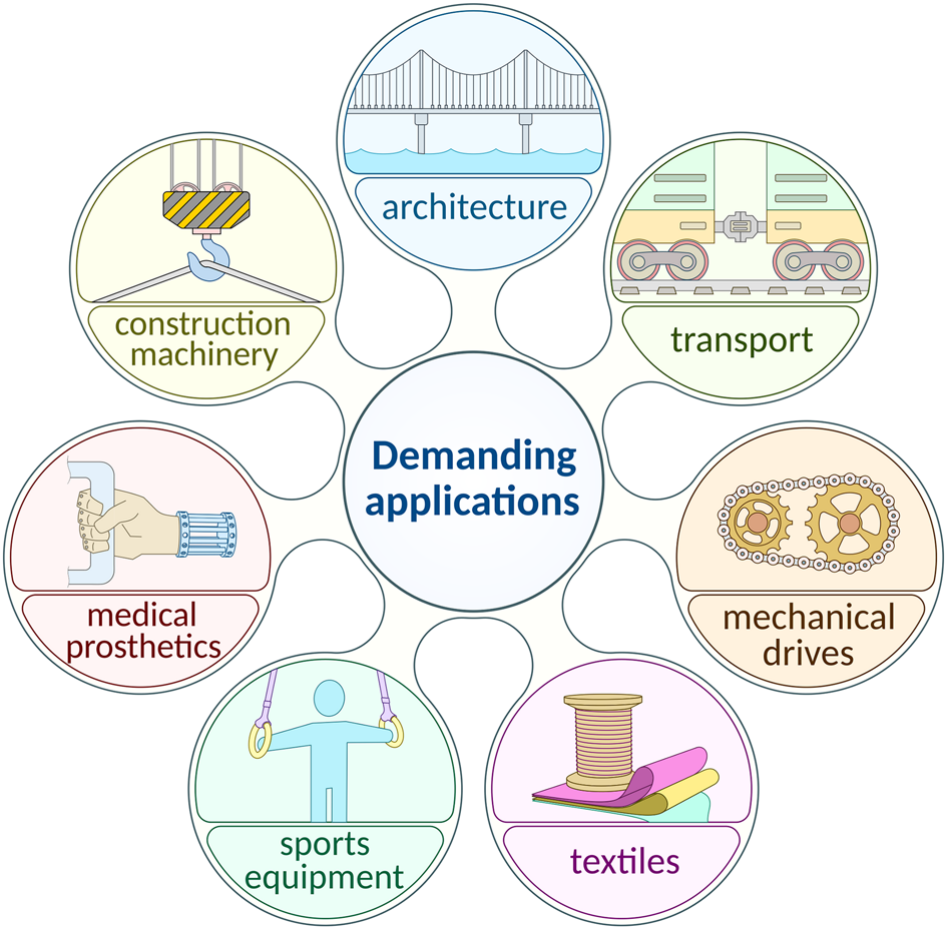
A basic chart of important applications of nondestructive analysis of mechanical properties.

Achieving the maximum strength of FDM parts involves not only choosing the right structural material but also carefully optimizing the part topology and parameters of additive manufacturing^41,42^. Therefore, the search and analysis of correlations between the material type, printing parameters, characteristics of the destruction process, and strength of printed parts for FDM technology is an urgent task. In the present study, we demonstrated that thermal imaging provides an easy-to-use efficient opportunity for the analysis of mechanical stress effects. Nondestructive analysis, observation at a distance (contactless) and good localization are important advantages of thermal analysis (Fig. 1). Importantly, connection with video streaming makes it possible to assess the mechanical stress effect in real-time observations if a rapid algorithm for data treatment is available. This became possible with the development of such a powerful and versatile data analysis tool as artificial neural networks^43^.

It is evident that the field experiences a very high rate of data acquisition^44–46^. These data require special approaches towards processing. Machine learning (ML) algorithms have revolutionized the area, making the automated extraction of relationships in data possible. Depending on whether correct labels are known or not, an algorithm falls into supervised or unsupervised machine learning groups^47,48^. This distinction here is not very strict, as newer methods were developed for situations where one has large amounts of unlabeled data and low amounts of data with labels. Another large area of machine learning is reinforcement learning, where the objective is to train an agent to perform actions, given observations and the state, to maximize the expected reward. The typical ML process includes four steps: creating a dataset, feature extraction, model training, and testing. The feature extraction process is often considered the most creative part of the data science process and is now usually performed by the algorithms themselves (for example, convolutional neural networks)^49^. It is important to note that in cases of volumes of data that cannot be processed on one machine, one needs to create a separate infrastructure (a Hadoop cluster, for instance)^50^, which becomes another step in the process.

The use of machine learning has already shown its efficiency in conjunction with additive technologies^43,51,52^. Currently, there are some studies aimed at applying machine learning in the field of mechanical testing of plastic and metal samples obtained using additive technologies^53–59^, including the manufacture of reactor parts^60^. Possible applications can be separated into three groups: a design for additive manufacturing, the manufacturing process itself, and search for structure–property/printing parameter-property relationships^43^. In design, researchers usually try to minimize the difference between expected and manufactured objects, create new materials, or automatically design the model to meet specific characteristics^61^. During the manufacturing process, one would need to control the parameters of a printer and check for defects. After that, to improve the quality of the product, relationships between printing parameters, structure, or other conditions and desired properties should be constructed.

Despite the presence of works focused on modeling thermographic data with neural networks^62^, there is no example usage of neural networks for fracture prediction in thermal imaging analysis. Therefore, here, we primarily focus on the task of fracture prediction from thermal images using neural networks, which is particularly important for industrial applications: analysis of the state of materials during their lifetime and prediction of possible destruction of constructions^63^. To do that, we aimed to develop a hardware platform for data acquisition along with software part to perform automated fracture prediction. For this work, we use 3D printed samples, showing the applicability of the approach for newly emerging technologies.

This work describes an efficient algorithm for acquiring large amounts of mechanical test data with thermographic video monitoring. The hardware part includes the universal testing machine and the thermographic camera connected to the single-board computer to improve the quality of the acquired data. The software part allows plotting time-resolved heatmaps, automated data analysis, and constructing correlations in data. As a result, we show a proof-of-concept for fracture time prediction.

## Results and discussion

### Hardware and software design for coupling mechanical tests and thermographic analysis

To couple multiple different analysis methods, one needs to carefully design both the hardware and software parts. Each test includes three stages: printing, testing, and analysis parts.

**Figure 2:**
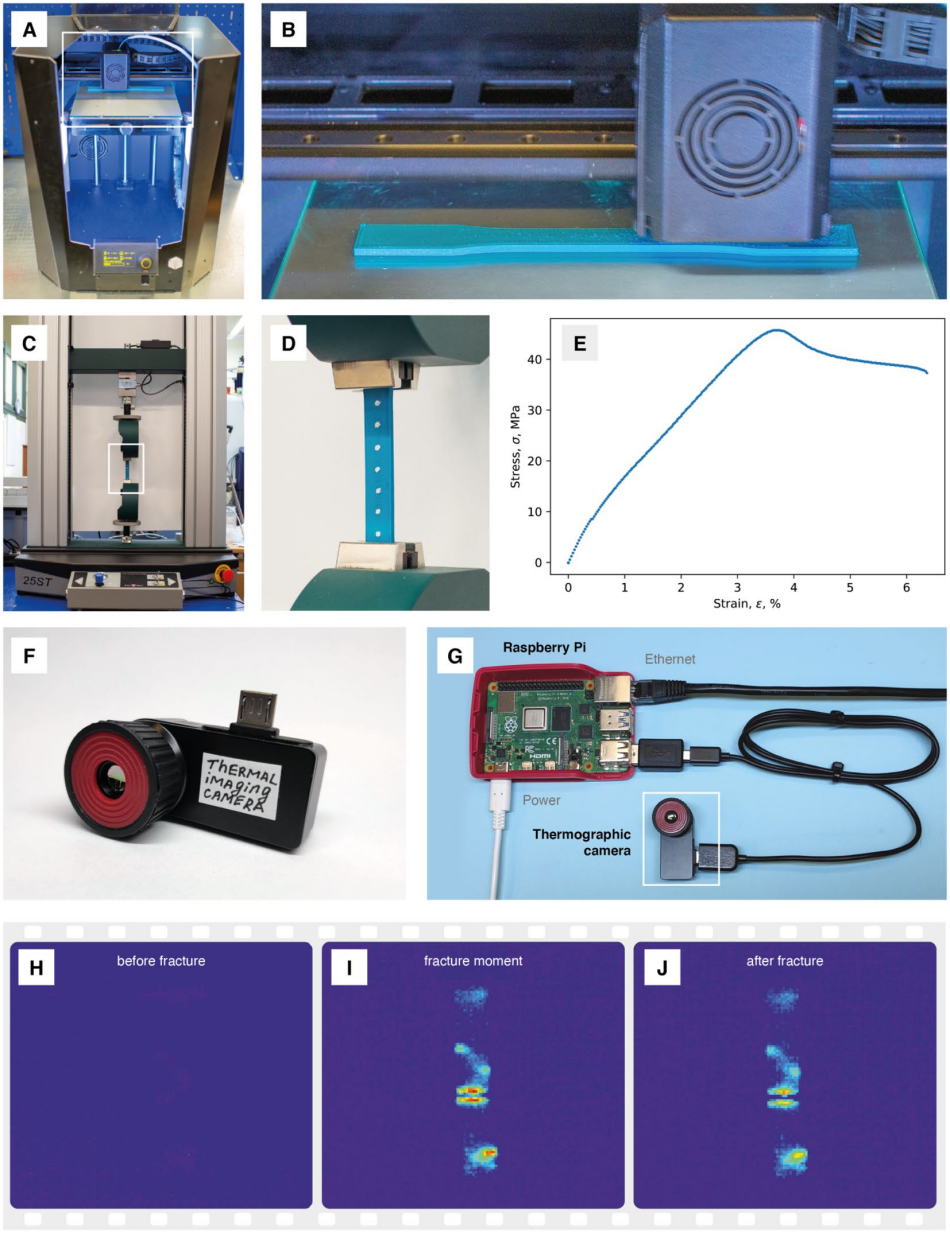
Overview of the approach for 3D printing, tensile and thermal analysis, and data acquisition. (**A**) Desktop FDM 3D printer Picaso X-Pro used for plastic specimens printing; (**B**) printing process of one of the plastic specimens; (**C**) general view of the universal testing machine Tinius Olsen 25ST used for tests of tensile strength; (**D**) one of the plastic specimens clamped in the grips of the machine and dotted for strain analysis; (**E**) stress–strain curve for one of the specimens; (**F**) Seek Thermal Compact Pro infrared camera for thermographic analysis of the destruction process; (**G**) infrared camera connected to Raspberry Pi compact computer for data collection and transfer; (**H**–**J**) thermograms of the plastic specimen during the tensile test: (**H**) before destruction, (**I**) destruction moment and (**J**) after destruction.

The printing part is performed by the FDM printing method. It is the most widespread method for obtaining 3D products of various shapes and configurations. To print a model, a 3D model must be designed first. This is done using CAD software. The next step is the preparation of the model for printing. Slicer programs allow variation of the mechanical and strength properties of materials by changing the density of the part infill, layer thickness, printing speed and other various characteristics. Finally, to print the model, we used an FDM 3D printer, characterized by high operating criteria, almost identical to industrial equipment (see Fig. 2A,B).

Testing the strength properties of various objects for all kinds of loads, such as tensile, compression and bend testing, is essential to many areas of human production and engineering. One of the most common types of loading is tensile testing (see Fig. 2C–E). As an example, all kinds of architectural objects (for example, bridges), medical devices (such as prostheses), and even fabric textiles experience this functioning under operating conditions (see Fig. 1). To subject the obtained samples to this type of load, it was decided to use a universal testing machine, the parameters of which are described in more detail in the experimental part.

During testing, thermographic data are collected. In this work, this is done using a mobile thermographic camera connected to a board computer (see Fig. 2F,G). The hardware setup extends a previously published approach^64^. The data are collected during testing and well-integrated with other parts of the data processing pipeline. Finally, the data were analyzed by custom Python scripts.

Thermographic data consist of videos recorded by a thermal imaging camera in the infrared range. Each frame represents a particular moment during the thermographic test. Pixels in such frames have values of the current temperature of the object. The camera was calibrated by sampling points from the 0–100 °C range. These frames can be further processed with custom software and be a source for machine learning algorithms (see Fig. 2H–J).

### Tensile testing

For this research, the following materials were considered: polylactic acid (PLA), advanced polycarbonate (PC+), styrene-acrylonitrile copolymer (CERAMO), advanced polylactic acid (PLA+), acrylonitrile butadiene styrene (ABS), acrylonitrile butadiene styrene fiberglass-filled (ABS-GF), acrylonitrile butadiene styrene filled with carbon fiber (ABS-CF), polyethylene terephthalate glycol (PETG), high impact polystyrene (HIPS), and polylactic acid with wood fibers (WOOD).

**Figure 3:**
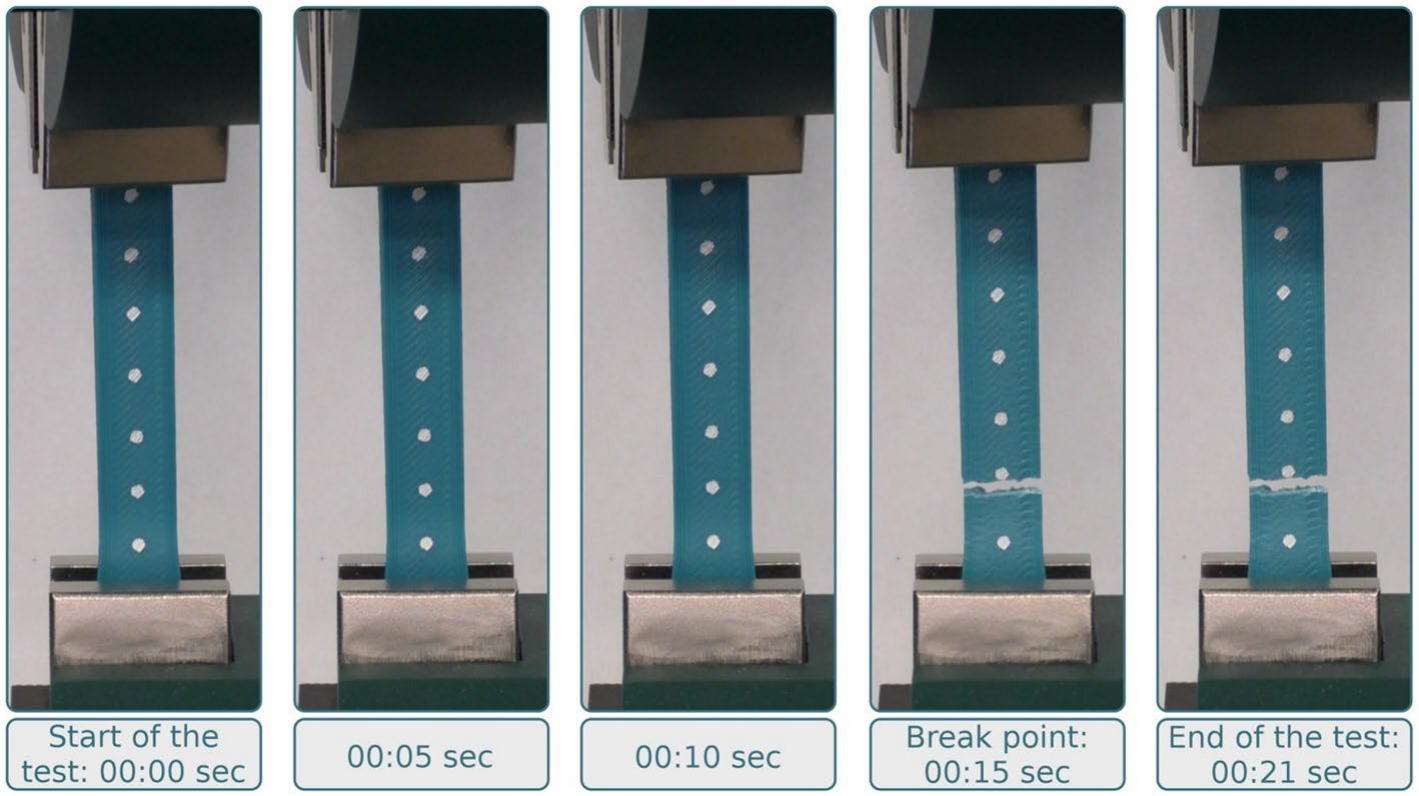
ABS-GF sample under the test conditions. The speed of movement of the upper traverse during the stretching of samples on a universal testing machine was 20 mm/min. The sample width was 13.3 mm, thickness was 3 mm, and estimated length was 80 mm. The width and thickness of the samples are taken from ASTM-D638-14 Type 1.

The samples were subjected to tensile tests using a universal testing machine. The machine performs the analysis with constant traverse speed, measuring the force needed. The test is carried out until the sample’s fracture (images of the samples under test conditions are provided in Fig. 3). Data obtained at this step include stress–strain curves, maximum strain/stress values, and the same values at the fracture moment. These values can then be used to calculate the strength characteristics of the materials.

To characterize the strength properties of the plastics under study, a number of tensile experiments were performed for each type of material. The main characteristics obtained using a universal testing machine are presented in Table 1 and Fig. S5. The data are averaged and ranked in order of decreasing strength.

**Table 1:** Physical and mechanical properties of plastics.

Plastics	Maximum tensile loads, kg	Deformation at ultimate strength, %	Displacement at the point of destruction, mm	Elongation at break, %	Tensile strength, MPa	Displacement to break, mm
PLA	257 ± 19	4.03 ± 0.32	4.26 ± 0.56	5.33 ± 0.70	63.1 ± 4.7	4.26 ± 0.56
PC+	245 ± 10	5.38 ± 0.44	4.97 ± 0.81	6.21 ± 1.02	60.2 ± 2.4	4.97 ± 0.81
CERAMO	213 ± 9	2.80 ± 0.15	2.24 ± 0.12	2.80 ± 0.15	52.5 ± 2.1	2.24 ± 0.12
PLA+	213 ± 9	3.49 ± 0.21	4.46 ± 1.28	5.58 ± 1.60	52.3 ± 2.3	4.46 ± 1.28
ABS	188 ± 3	3.76 ± 0.17	4.98 ± 1.04	6.23 ± 1.31	46.2 ± 0.7	4.98 ± 1.04
ABS-GF	170 ± 6	3.66 ± 0.31	3.24 ± 0.30	4.05 ± 0.38	41.7 ± 1.4	3.24 ± 0.30
ABS-CF	161 ± 10	3.58 ± 0.18	4.36 ± 0.44	5.45 ± 0.55	39.7 ± 2.6	4.36 ± 0.44
PETG	149 ± 26	4.93 ± 0.62	5.00 ± 1.05	5.81 ± 1.42	36.6 ± 6.6	4.65 ± 1.13
HIPS	105 ± 3.9	2.31 ± 0.09	4.02 ± 1.00	5.02 ± 1.25	25.8 ± 1.0	4.02 ± 1.00
WOOD	77.7 ± 5.1	3.36 ± 0.39	3.00 ± 0.39	3.75 ± 0.49	19.1 ± 1.3	3.00 ± 0.39

To put that into perspective, the maximum tensile loads (kg) in decreasing order can be used to create the following strength series: PLA (257) > PC+ (245) >> CERAMO (213) ≈ PLA+ (213) > ABS (188) > ABS-GF (170) > ABS-CF (161) > PETG (149) >> HIPS (105) > WOOD (78).

The same range of strength characteristics can be described for tensile strength (MPa): PLA (63.1) > PC+ (60.2) >> СERAMO (52.5) > PLA+ (52.3) > ABS (46.2) > ABS-GF (41.7) > ABS-CF (39.7) > PETG (36.6) >> HIPS (25.8) > WOOD (19.1).

It should be noted that glass-filled and carbon-filled ABS show worse physical and mechanical properties than unfilled ABS. This relationship may be due to the presence of additional components in the composition. When printing a layered structure, the solidity of the product is reduced due to poorer adhesion and reduced mutual fusion of the layers, although the individual materials themselves are claimed to be more durable.

WOOD (wood fiber-filled PLA) has the worst tensile strength, which is explained by its reinforcement with short wood fibers, which prevent hardening and affect the increase in the porosity of the structure of the product. This material has a more decorative function and should not be expected to show superior tensile properties.

The 10 out of the 15 most consistent experiments for each individual material were plotted (Fig. S4). In each series of tests, five tests were removed due to the significant number of outliers, which is caused by the complexity of additively manufactured samples with a layered structure and the characteristics of the testing machine, namely, the strain gauge can sometimes provide incorrect data. Separate, not fractured layers are perceived by the strain gauge as an unfinished experiment, after which the test continues, but, in fact, the sample is sufficiently deformed. Additionally, sometimes the test is terminated, although the sample was not destroyed. These issues are easy to control and notice during the experiment. The selection of 10 experiments out of 15 attempts provides enough statistical averaging data.

To increase the strength of the finished filled product, one needs to carefully optimize printing parameters to reduce the resulting defects and study the effect of optimization on fiber position using microscopy techniques.

All construction materials can be subjected to simple fracture under the action of a static load, in our case under the action of tension, by a plastic or brittle mechanism.

### Thermal imaging for visualizing deformations

To obtain more insight into the processes occurring in the materials, we performed thermographic analysis. Figure 2I shows that at the fracture point, intense heating may be observed. Before that, materials behave differently: some heat very slowly just before the fracture, and some reduce the observed temperature. This cooling may be caused by the change in the material’s ability to reflect infrared radiation. After fracture, the heated polymer slowly cools to room temperature.

**Figure 4:**
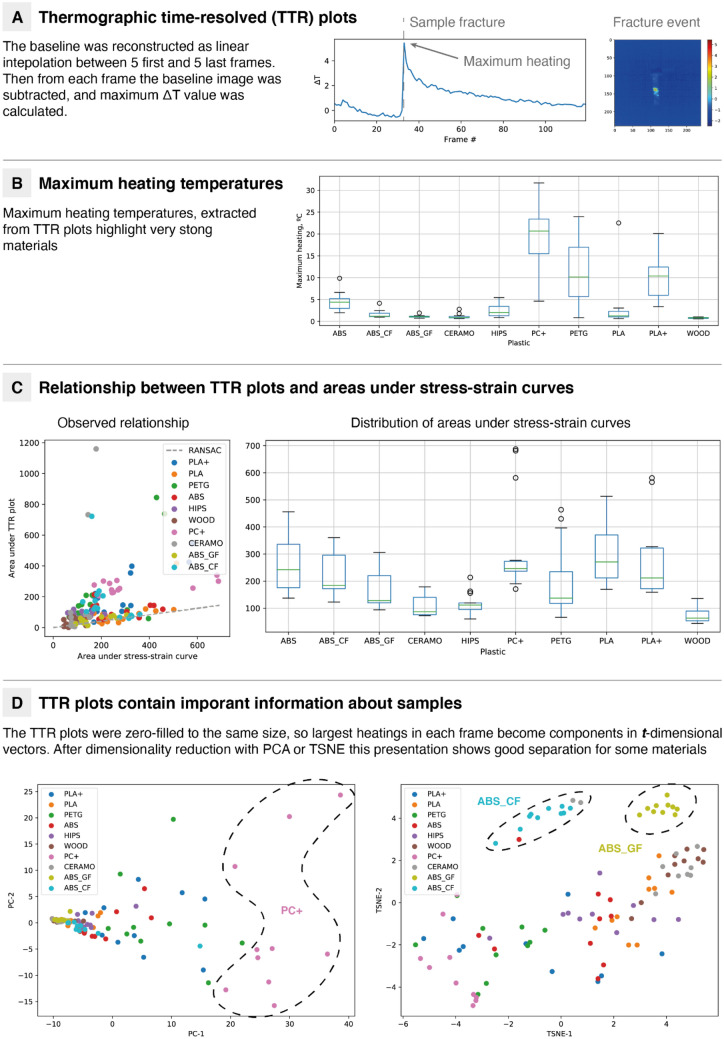
Overview of information available from thermographic videos. (**A**) describes thermographic plots and maximum heating temperatures. (**B**) Shows the distribution of maximum heating temperatures for different materials. (**C**) Observed relationships between thermographic and mechanical data. (**D**) Shows the use of entire curves for material characterization.

The observed behavior may be visualized as plots of the maximum frame heating (done via background subtraction, which is calculated as a linear interpolation between averages of the first and averages of the last frames) against time or a frame number (thermographic time-resolved plots, TTR plots). These relationships usually consist of a highly intensive peak, which corresponds to the material fracture event (Fig. 4A). Analysis of the thermographic imaging frame at the fracture moment enables observation of the exact place where a crack is being developed. Additionally, most of the materials show prefracture heating (although this might not be represented by TTR plots due to the low intensity of the signal) during the deformation process^65–67^. As tensile testing experiments do not result in fully elastic deformation, the process is irreversible, which means that some of the energy supplied must be released as heat.

In some materials, this relationship is not well pronounced (for example, WOOD). Others, such as PC+, show huge maximum heating with an average of 22 °C (Fig. 4B). PC+ is also characterized by special features, such as high temperature resistance (up to 110 °C), impact resistance and toughness, which makes this material an alternative to metals and characterizes the behavior shown in Fig. S5. Polycarbonate occupies a leading position in almost all physical and mechanical properties, apart from displacement at the point of destruction (shares leadership with ABS and PETG), maximum tensile loads and tensile strength, where PLA has a leading position, but the numerical difference between PLA and PC+ is small.

Heating temperatures also reflect some properties of the materials, in particular the total energy stored in the material during the trial. The total energy dissipation potential can be calculated from stain-stress curves as the integral of stress over strain^68^:

$$TE=\underset{\varepsilon =0}{\overset{{\varepsilon }_{max }}{\int }}\sigma d\varepsilon =\underset{t=0}{\overset{{t}_{max }}{\int }} \sigma \frac{d\varepsilon }{dt} dt={E}_{heating}+{E}_{other}=\underset{t=0}{\overset{{t}_{max }}{\int }}\underset{\left(x,y\right)\in I}{\overset{}{\iint }}(\rho z){C}_{p}\Delta Tdxdy dt+{E}_{other}\approx \underset{t=0}{\overset{{t}_{max }}{\int }}\underset{\left(x,y\right)\in I}{\overset{}{\iint }}(\rho z){C}_{p}\Delta Tdxdy dt\approx \underset{t=0}{\overset{{t}_{max }}{\int }}TTR(t) dt,$$

where $\rho$ is the density of the material, $z$ is the thickness of the sample, $\epsilon$ is the deformation, $\sigma$ is the stress, ${C}_{p}$ is the heat capacity, and $\Delta T$ is the difference from the baseline temperature. The corresponding integral may be approximated as an area under the TTR plot, assuming a similar heating pattern during the trial and a small value of ${E}_{other}$. This relationship is indeed observed for some materials (Fig. 4C). Maximum heating temperatures were also calculated, but the relationships were not well pronounced (Table S3).

To further highlight the differentiating ability of the TTR plots, corresponding data were vectorized, and dimensionality reduction analysis was applied. Converting these values into vectors and zero-filling to the same length, a good representation for the material may be obtained (Fig. 4D). For instance, in principal component analysis (PCA) plots, PC+ can be successfully separated. In t-distributed stochastic neighbor embedding (TSNE) plots, two clusters of ABS-CF and ABS-GF are observed.

Thus, it can be easily noted that thermographic observations provide another important dimension for mechanical test data. They can be used to characterize accumulated energy during the trial and possibly differentiate materials from each other. The amount of information present in TTR plots suggests that full thermographic videos are well suited for automated analysis with machine learning methods.

### Fracture time can be successfully predicted using ML models

Fracture prediction by thermal analysis, if possible, would be one of the most practically applicable tasks in materials science. Many real constructions, such as buildings or bridges and mechanisms (such as bicycle chains), experience several forces. In some cases, they can lead to material fracture. Therefore, identification of conditions, probably leading to fracture, and prediction of time before the fraction is a crucial task for efficient monitoring.

Modeling of the fracture process was studied extensively. One of the main approaches is the finite element model (FEM). The simulations have a high computational cost, so machine learning approaches were developed. Examples include crack propagation predictions for brittle materials^69–71^ and graphene^72^.

Input data such as initial crack geometry or even current deformation and stress may be used efficiently to predict the fracture time. Unfortunately, in a real setting, these data are not available. Additionally, the exact type of material may be known, but the development of an algorithm not requiring information about the material as input would increase the applicability of the technology. We used thermographic camera image series as input data for the neural network regression models to solve the problem.

**Figure 5:**
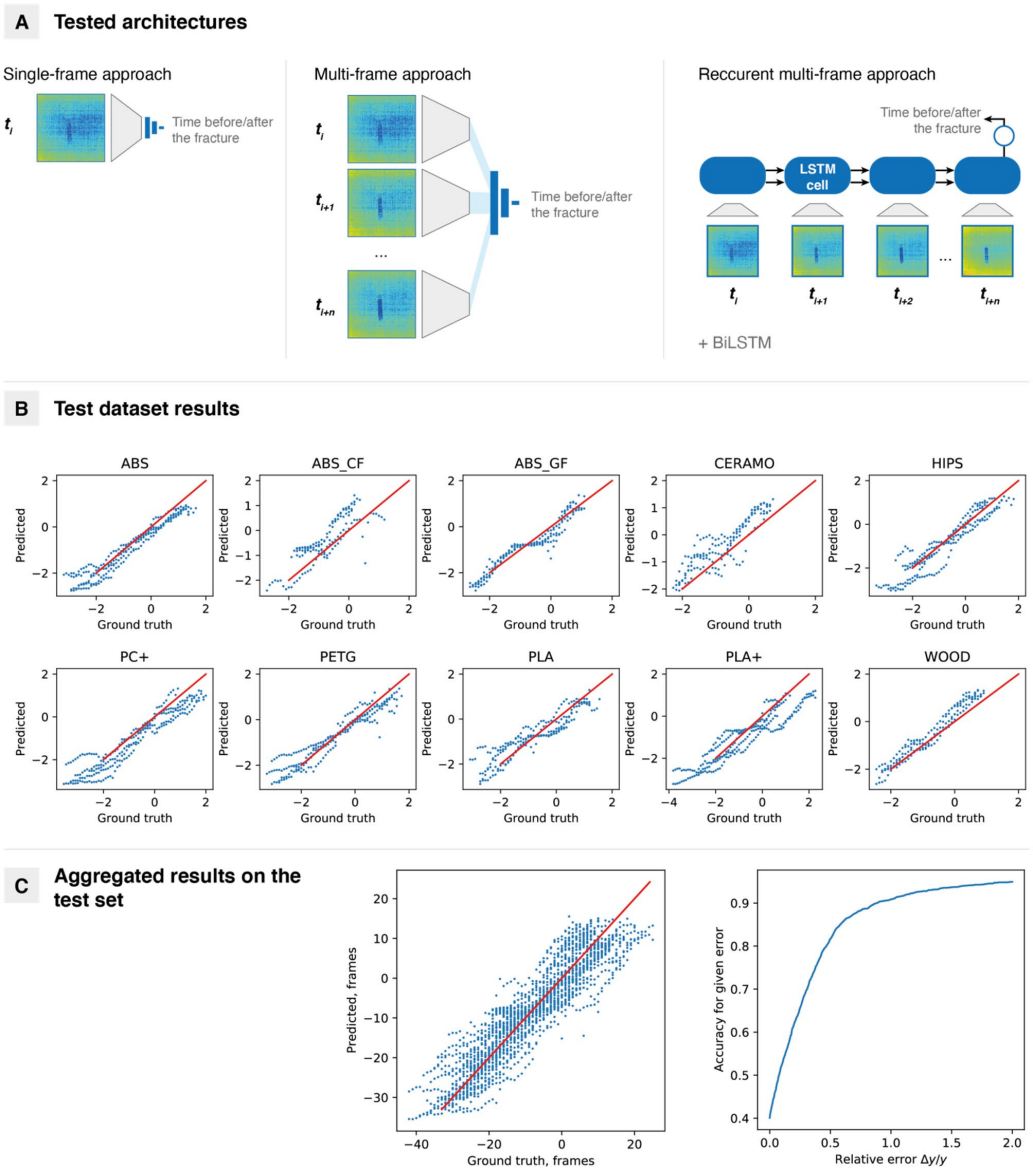
Neural network analysis of thermographic images. (**A**) Comparison of architectures; (**B**) Results for the best model by material; (**C**) Aggregated results on the test set for the best model. Model—bidirectional LSTM model with an embedding size of 256 elements. Ten consecutive frames were used for prediction. The hidden size is equal to 256. Each batch contained 200 sequences.

Three types of architectures were proposed: single-frame, multiframe, and recurrent multiframe models (Fig. 5A). They map images into high-dimensional space using convolutional encoders but differ in further processing. In the single-frame approach, only one image was used, and then the resulting vector was passed into a fully connected neural network (FCNN). This may be extended to the multiframe case if embeddings for each frame were concatenated and then passed into the FCNN. Finally, the temporal nature of the image sequences may be captured by recurrent neural networks to be more specific, long short-term memory neural networks (LSTMs). The image embeddings were passed from the convolutional encoder as well, and the last LSTM cell hidden state was passed into the FCNN. In the bidirectional case, the corresponding hidden states were concatenated and again passed into the FCNN.

The training was performed with the MSE loss function^47^ ${\mathcal{L}}_{MSE}$ (see more details on training in the “Methods” section):

$${\mathcal{L}}_{MSE}\left(y,\widehat{y}\right)=\frac{1}{{n}_{samples}}\sum_{i=0}^{{n}_{samples}}{\left({y}_{i}-{\widehat{y}}_{i}\right)}^{2},$$

where $y$ and $\widehat{y}$ are the ground truth and prediction vectors, respectively, and ${n}_{samples}$ is the number of elements in the vector.

We predicted both times before the fraction and after to increase model robustness and provide more training data.

Neural networks successfully learn the relationships, but a lack of data causes severe overfitting. To reduce its effects, batch sizes were reduced, and encoder architectures were simplified as much as possible. Batch normalization layers played the same role^73^.

As shown in Table 2, the recurrent approach shows the best results across the described architectures: 0.33 MSE loss (lower is better) in a single-frame case against 0.23 in a recurrent case. This is equal to 72% and 82% accuracies at the 50% value difference level. Generally, there is no significant impact of embedding vector lengths on the model’s performance, although the best model had 256 elements in the embedding vector with an MSE loss of 0.23 (mean absolute error of 0.37, root mean squared error of 0.48). Moreover, even with the same number of frames and the same length of the embedding vector, recurrent models outperform multiframe models. Bidirectional models show considerable improvement for lower dimensions. Generally, better performance is observed for a larger number of input frames in both multiframe and recurrent cases (from 7 to 10 frames). Comparison with training metrics shows a significant degree of overfitting (Table S7); however, the use of validation and testing datasets reduces the bias and helps to choose the best model based on its ability to generalize towards training data. Moreover, adding more data is highly likely to increase the performance of the model.

**Table 2:** Results for the architecture search.

Architecture	Best MSE loss	Accuracy		
		5%	10%	50%
**Single-frame**				
256	0.3664	0.28	0.31	0.49
1024	0.3278	0.41	0.45	0.72
**Multi-frame**				
256	0.2633 (9 frames)	0.42	0.47	0.80
1024	0.2861 (10 frames)	0.41	0.45	0.77
**Recurrent**				
256	0.2605 (7 frames)	0.47	0.52	**0.84**
256*	**0.2310 **(10 frames)	0.47	0.52	0.82
1024	0.2523 (7 frames)	**0.49**	0.52	0.82
1024*	0.2775 (10 frames)	**0.49**	**0.55**	**0.84**

Mean squared error loss values for the normalized target (the same as in Fig. 5) and corresponding accuracies for 5%, 10%, and 50% deviations from the target value are provided. For multiframe and recurrent models, the number of input frames is shown. The best values are shown in bold.

*Bidirectional; the best results are shown in bold.

As a result, a neural network for fracture time prediction was developed (see Fig. 5B for results by material and Fig. 5C for aggregated results). The best model uses a convolutional encoder to generate frame embeddings and then passes them into the bidirectional LSTM network. Moreover, the model is not aware of the exact material used in the experiments, where corresponding input sequences were generated.

As no explicit modeling of the processes that occurred during the mechanical tests was made, the models can be learned purely from experimental data. Therefore, the main advantage of the approach is that the network can learn effects, which may be hard to model, and a single architecture can be used for various types of samples and objects. Moreover, predictions can be made very rapidly, reaching real-time speed.

However, the interpretability of the models is a significant limitation. As seen from the analysis of output gradients with respect to the input images, at some timesteps, the network focuses on either the bottom part of the sample (Fig. S8) or at the point of developing (10th frame at Fig. S8) or already developed (Fig. S9) fracture. Unfortunately, standard approaches such as FEM provide much more insight into internal physical processes.

### A layer of another polymer can be used as a thermographic marker

The performed experiments show that materials have different temperature dynamics during mechanical testing. In some cases, it reduces the quality of the fracture time prediction. For instance, the model performance is the worst in the case of carbon-filled ABS plastic. It was proposed that the quality of the analysis can be significantly enhanced if another layer of more “thermally active” polymer was placed on top of the main materials. These experiments were performed for layers of PLA, multiple types of adhesive tape and glues.

These materials were applied to the front and back surfaces of the test sample. Experiments of such designs were carried out on a universal testing machine using a thermal imaging camera. The obtained strength properties are presented in Table S6 and Fig. S6.

According to Fig. S6, such a "modification" of carbon-filled plastic insignificantly affects the physical and mechanical parameters, which indeed confirms the feasibility of using this method to improve the temperature dynamics.

Next, consider the value of the maximum heating of ABS-CF plastic in combination with materials that improve the graphical picture of prediction. Table S6 shows that some materials increase the maximum heating value of ABS plastic reinforced with carbon fiber, and some materials, on the contrary, decrease the value, which is associated with the nature of the material used and the location of this material on the sample surface.

**Figure 6:**
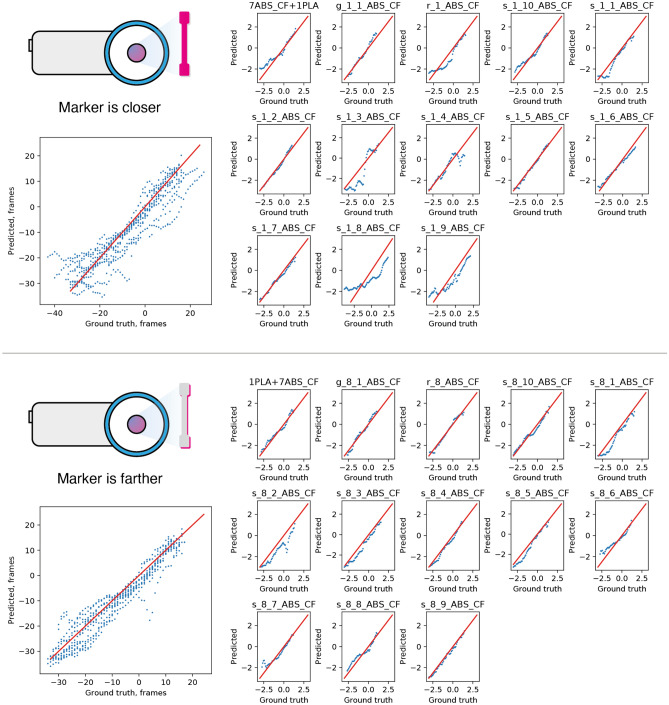
Results for different potential thermographic markers after neural network analysis.

Finally, corresponding thermographic videos were fed into the neural network described in the previous section (Fig. 6). Most potential markers indeed enhance the thermal behavior of the materials. However, surprisingly, measurements performed on the other side of the sample are even better. This effect is likely caused by two reasons: the thermal behavior of markers is new for the neural network, so predictions are worse, which is expected. However, keeping two parts of the sample connected during the test increases its thermal activity and prolongs the crack growth process.

### Extension to the multisample case

Finally, attempts were made to analyze whether the described approach is applicable for more complex structures. As an example, an experiment for tensile testing of four samples at once was designed (Fig. S7). In total, five experiments were recorded, and their physical and mechanical characteristics were calculated (Table S6).

The videos were then fed into the neural network, which was retrained to operate on an image crop containing only the sample without background. Each of the five videos was cropped into four different videos for each sample, which were then fed into the neural network.

**Figure 7:**
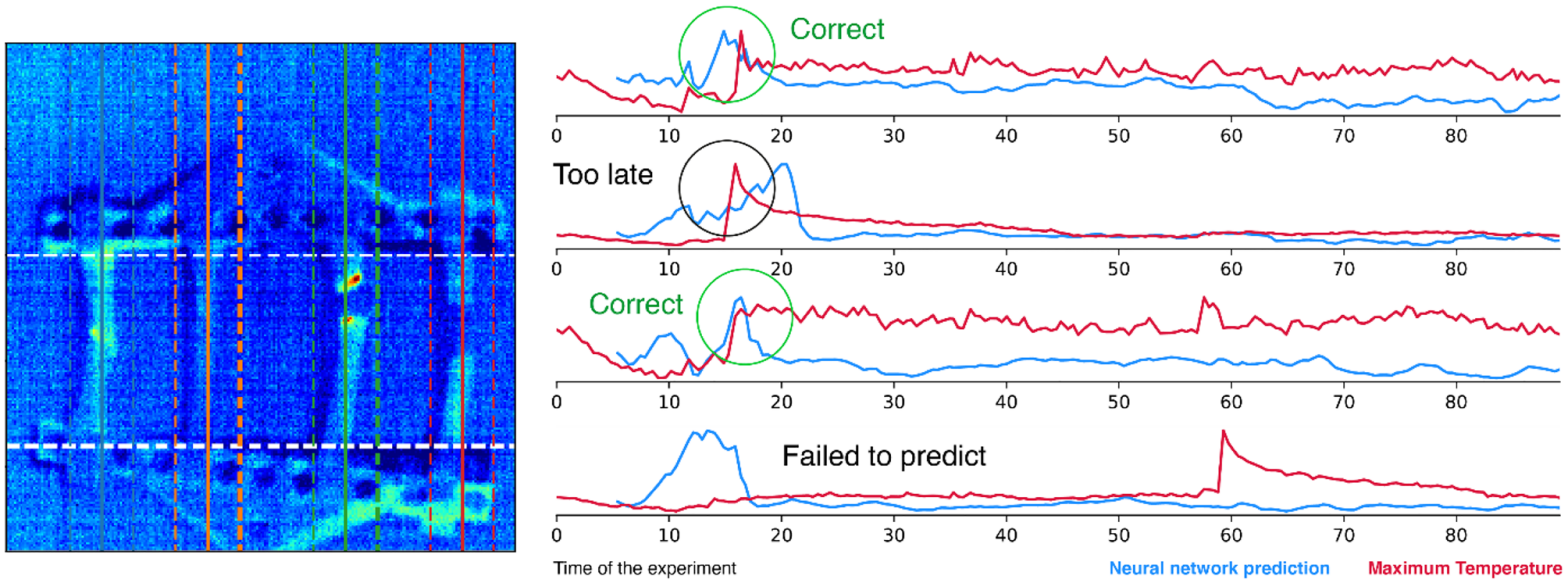
Results of neural network predictions for the case of four samples tested at once.

Although some signal was observed, it was hard to reproduce. For example, for one of the videos (Fig. 7), only two fracture events were predicted correctly. For the fourth sample, the event was not predicted at all, while for the second sample, the event occurred earlier. In some cases, the trend of the network prediction agreed with the experimental observations.

In our opinion, this is due to the influence on the output data of the built-up hold-down construction and the ripple deformation of individual samples of each test series, due to the unique character of discontinuity: synchronistic deformation of the first two dog-bones to the right or to the left relative to the center of the built-up construction, deformation of the first dog-bone in different areas of the construction, and different times between sample fractures, which, as a whole, led to the displacement of the construction relative to the vertical plane in different directions during testing. At the same time, the observed results suggest that correct prediction is possible and might be accomplished with changes in the algorithm.

This opens a new way to improve the algorithms: they should be trained on a wider variety of systems to improve the generalizability of the models. This work made a step forward in this direction, implementing an algorithm for automated fracture prediction.

## Conclusions

In conclusion, thermographic data can be successfully used to study samples during mechanical tests. The thermal behavior of materials is another way to characterize materials and distinguish them from each other. Underlying physical reasons for such behavior were analyzed, and correlations were drawn. A new characteristic of the material’s thermal behavior—maximum heating—was introduced and calculated for ten materials (ranging from 0.80 to 19.65 °C).

As a result, the data were subjected to machine learning analysis: a neural network for fracture time prediction was developed (with MAE of 0.37 and MSE of 0.23). Comparing the model performance in cases of different materials, an approach to increase the thermal activity of the polymer was developed.

As a thermographic marker, Type 6 should be selected, as it shows the highest maximum load (182 kg), tensile strength (44.7 MPa) and maximum heating (10.60 ºC). Notably, fixation of the material should be carried out on the back of the sample to achieve the highest output characteristics. This behavior might be explained by the adhesive changing the pattern of the crack development process, which results in slower heat release rates.

Structural materials, mechanisms, building objects, and various items during operation in many fields of activity require special attention in predicting the long-term service life and their quality; therefore, the provided results confirm the importance of thermographic measurements in mechanical tests and their high potential in moving towards efficient monitoring of constructions and mechanisms during their lifetime.

## Methods

### 3D printing of test samples

All samples were made by the FDM method (fused deposition modeling) using a personal desktop 3D printer Picaso Designer X-Pro (see Fig. 2A) in 10 pieces for each type of plastic.

A typical tensile specimen (dog-bone) was made according to standard ASTM D638-14 Type I (Fig. S1A,B). The G-code was generated by Polygon X software. During printing, the plane of the samples was parallel to the XY plane of the printer build plate (Fig. S1C,D).

In all cases, printing was performed using a steel nozzle with a diameter of 0.5 mm, an extrusion width of 0.55 mm, a density of internal filling of samples of 100%, and a retraction value of 1 mm. For all materials except ABS-CF and PC+, a special adhesive was used to improve the adhesion of the part to the surface of the printer build plate. In the case of polycarbonate, the adhesion of the material to the glass was excessive, resulting in damage to the surface of the printer build plate when the finished part was removed. Therefore, for PC+, paper tape was used as the coating of the printer build plate. The temperature parameters of additive manufacturing for all materials studied in the work (Fig. S1E) are shown in Table S1.

### Tensile testing and thermographic analysis

The tests were carried out with the Tinius Olsen 25ST universal testing machine (maximum capacity 25 kN). The load weighing system of the testing machine exceeds the requirements of the following standards: ASTM E4, ISO 7500-1, and EN 10002-2; the strain measurement system of the machine exceeds the requirements of the following standards: ASTM E83, ISO 9513, EN 10002-4. During the tests, self-tightening wedge grips and a load cell with a maximum measurable load of 25 kN were used. The load cell accuracy in tensile measurements is 0.5% in the load range from 50 N to 25 kN. The tests were carried out at a moving crosshead speed of 20 mm/min. The results processing and analysis were carried out using the “Horizon” software package.

The obtained strength characteristics of unfilled plastics are comparable with the values provided in the database^74^ containing the characteristics of polymer materials from many manufacturers (Table S3).

Thermographic analysis was performed using a Seek Thermal Compact Pro thermal imaging camera with a highly sensitive infrared sensor. The measuring range of the temperature of this camera lies in the range from − 40 to + 330 °C. The transfer of the images obtained during shooting is carried out with a frequency of 9 fps. The camera was installed in front of the testing specimen to capture the thermal effects of the sample destruction and connected to a microcomputer.

#### Multisample case

To see whether the described approach can be extended to cases where multiple samples are tested simultaneously, a built-up hold-down construction was developed. As a result, 3D printed specimens were fixed in a testing machine by mechanical steel clamps.

The central part of the setup is a frame made of steel elements with holes assembled with bolts and nuts. It was attached to a 3D printed base (Fig. S7). Four samples were placed between two parts of the construction, fixed with bolting, and tightened with a wrench. It is important to fix the samples with force such that their deformation is minimal. Deformations lead to stress concentrations and consequently result in the destruction of the samples during testing.

As thermal effects were much harder to observe, the camera was connected to a laptop with a higher bandwidth connection than the Raspberry Pi board. However, the software and the experimental procedure were kept the same as in the previous experiments. In total, five series of tests were acquired. In every case, fracture occurred in the working section area. The results of the physical and mechanical experiments are presented in Table S6 for each set of tests.

### Data acquisition

The original software for the thermographic camera used does not give enough flexibility for automated analysis due to temperature scale constraints. There are two options. The first is to fix the scale by the lowest and the highest temperature and hope that the temperatures will not exceed them during the experiment. The second would mean keeping the scale adaptive, but in every frame of the resulting video, one would need to recognize all the text reconstructing the scale and temperatures according to the colors. The latter option is the separate task and increases the pipeline complexity dramatically.

Therefore, we developed another approach based on direct parsing of the camera’s output, connected by USB to the Raspberry Pi single-board computer (Fig. S2). The image acquisition relied on already available projects^75^ and was packed into a web application written using the Flask Python framework. The operator has two options: to record one image or to start video recording. The raw data are saved locally on the Raspberry Pi and then can be transferred via SFTP.

Raw data consist of numbers, which are linearly dependent on the object’s temperature. Therefore, we performed a calibration of our particular device. The resulting curve (Fig. S3) shows a high R^2^ value (0.993), which indeed confirms the linearity. All images were then decoded accordingly.

Another significant problem in data processing is the presence of background noise and brightening caused by other objects. It was solved by subtracting averages of multiple first frames from every from in a video.

As a result, we developed a convenient way to acquire and process large amounts of thermographic data. It includes a single-board computer to parse data from the thermographic camera directly and a web application with a user-friendly interface to operate it.

### Machine learning analysis

Data were processed with the NumPy^76^ and Pandas^77^ packages and plotted with Matplotlib^78^. Scikit-learn^79^ implementations of PCA and TSNE were used in dimensionality reduction. Neural networks were implemented using the PyTorch Python package^80^ in the PyTorch Lightning framework^81^. Models were trained with the Adam optimizer^82^.

Convolutional encoders had the following structure: *conv*(1, 2, 3), *ReLU*, *batchnorm*, *conv*(2, 4, 3), *ReLU*, *batchnorm*, *maxpool*(2), *conv*(4, 4, 3), *ReLU*, *batchnorm*, *conv*(4, 4, 3), *ReLU*, *batchnorm*, *maxpool*(2), *conv*(4, 4, 3), *ReLU*, *batchnorm*, *maxpool*(2) (optional), *conv*(4, 4, 3), *ReLU*, *batchnorm*, *maxpool*(2) (*conv*(***a***, ***b***, ***c***) = convolutional layer with input channels, ***b*** output channels and (***c***, ***c***) – window size). The fully connected regressor applied a linear layer to the vector with shape (16), then ReLU, and finally, a linear layer to one number. Selection of the activation function was performed in the initial architecture search and was based on the metrics of the model.

Long sort-term memory neural networks are a type of recurrent neural network and perform the following transformation for each input^83^:

$${i}_{t}=\sigma ({W}_{ii}{x}_{t}+{b}_{ii}+{W}_{hi}{h}_{t-1}+{b}_{hi})$$

$${f}_{t}=\sigma \left({W}_{if}{x}_{t}+{b}_{if}+{W}_{hf}{h}_{t-1}+{b}_{hf}\right)$$

$${g}_{t}=\mathrm{tanh}\left({W}_{ig}{x}_{t}+{b}_{ig}+{W}_{hg}{h}_{t-1}+{b}_{hg}\right)$$

$${o}_{t}=\sigma \left({W}_{io}{x}_{t}+{b}_{io}+{W}_{ho}{h}_{t-1}+{b}_{ho}\right)$$

$${c}_{t}={f}_{t}\odot {c}_{t-1}+{i}_{t}\odot {g}_{t}$$

$${h}_{t}={o}_{t}\odot \mathrm{tanh}({c}_{t})$$

Here, $\odot$ is the Hadamard product, and $\sigma$ is a sigmoid function.

Therefore, at each step, the hidden states ${h}_{t-1}$ at time $t-1$ are converted into output hidden states ${h}_{t}$, and the same happens with cell states ${c}_{t}$. The process is controlled by gates $i$, $f$, and $o$, which are called input, forget, and output gates, respectively, $g$ is the cell state. Network weights are represented as $W$ matrices.

For the best recurrent model, training was performed for 250 epochs with a batch size equal to 150. Ten consecutive frames were analyzed.

## Supplementary Information

Supplementary Information.

## Data Availability

All necessary data are provided in this article and supplementary information.
