# Supplementary material for: Integration of thermal imaging and neural networks for mechanical strength analysis and fracture prediction in 3D-printed plastic parts
Source: Sci Rep. 2022 May 27;12:8944. doi: 10.1038/s41598-022-12503-y (PMC9142534; doi:10.1038/s41598-022-12503-y)
Supplement: Supplementary file 1 — Supplementary Information. [file 41598_2022_12503_MOESM1_ESM.pdf]

Supporting information
for the article:

Integration of thermal imaging and neural networks for mechanical strength analysis and fracture prediction in 3D-printed plastic parts

Daniil A. Boiko, Victoria A. Korabelnikova, Evgeniy G. Gordeev, and Valentine P. Ananikov*

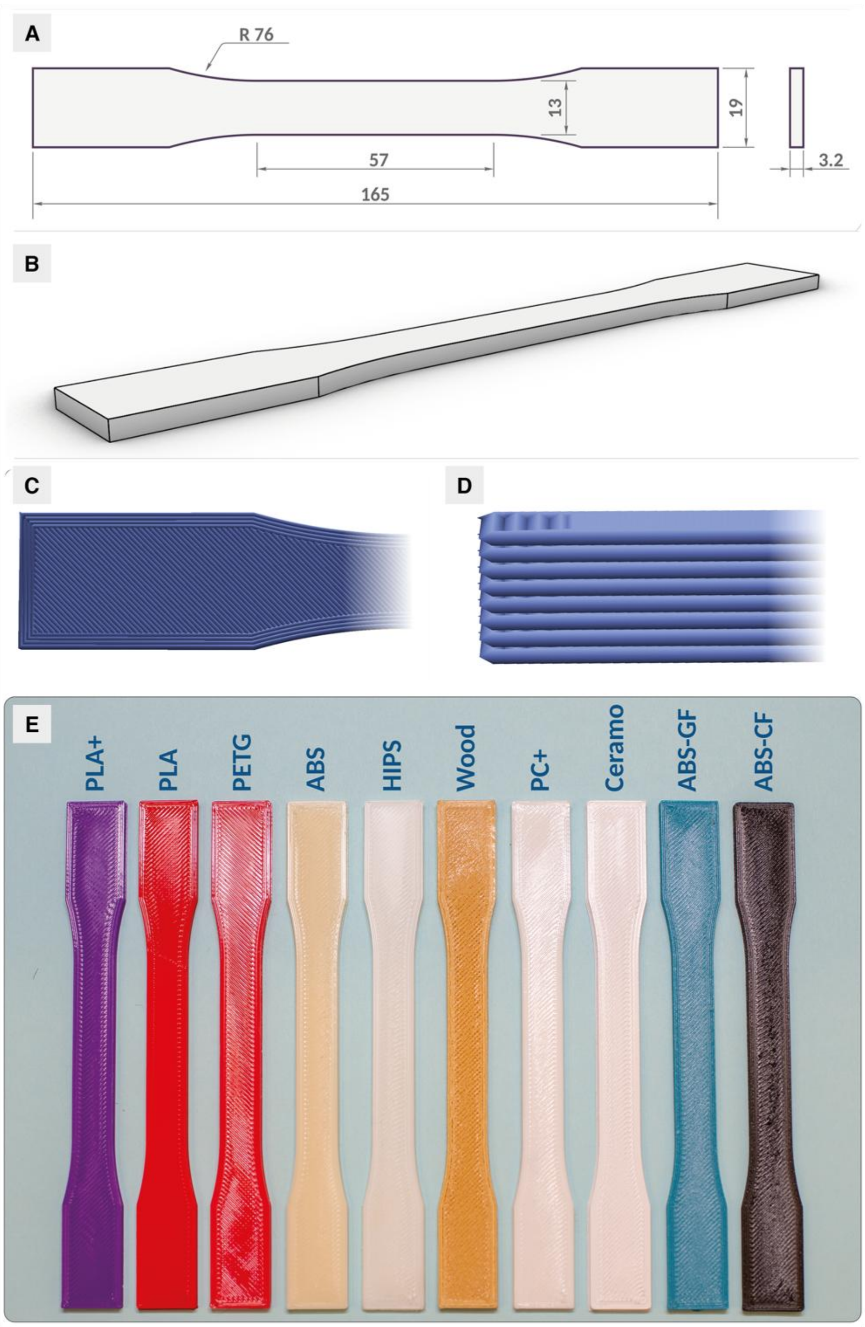

Figure S1. **(a)** Specimen Type I dimensions according to ASTM D638−14 standard, **(b)** 3D view of the specimen Type I model used for 3D printing and testing, **(c)** G-code visualization for specimen from top view illustrating perimeters and internal infill, **(d)** G-code visualization for specimen from side view illustrating layers count and arrangement, **(e)** ready-to-use specimens made of different thermoplastics.

Table S1. Print temperature parameters and extrusion multiplier values for all materials.

	PLA	PLA+	PETG	ABS	HIPS	WOOD	PC+	CERAMO	ABS-GF	ABS-CF
T nozzle, °C	220	220	240	245	245	220	295	250	280	280
T bed, °C	70	70	80	100	95	80	110	100	100	100
Extrusion multiplier	1.00	1.00	1.00	1.00	1.00	1.00	0.98	0.98	1.00	1.00

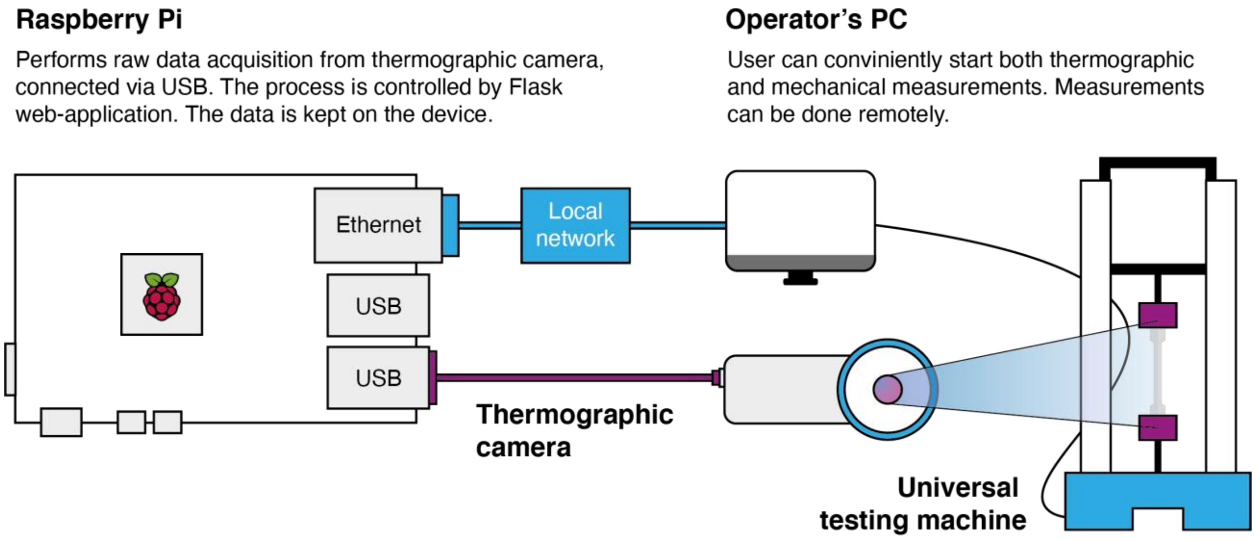

Figure S2. Scheme of the data acquisition approach.

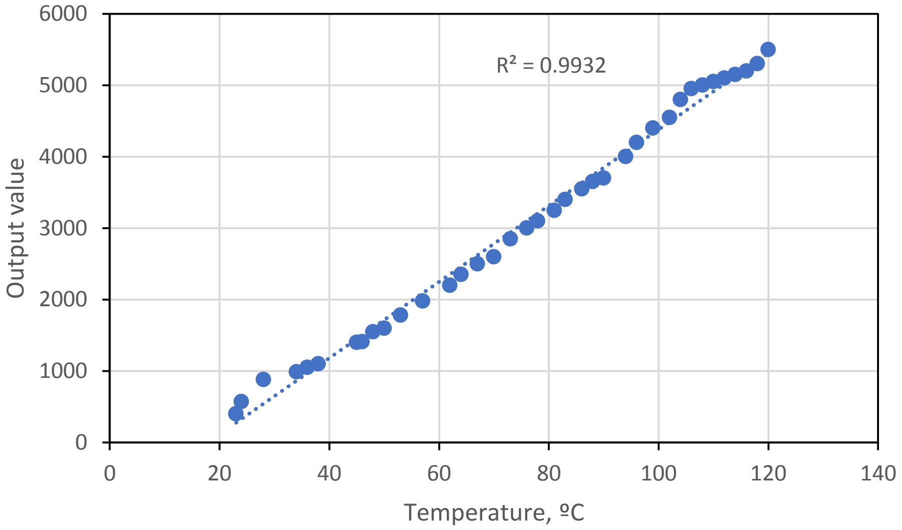

Figure S3. Calibration curve

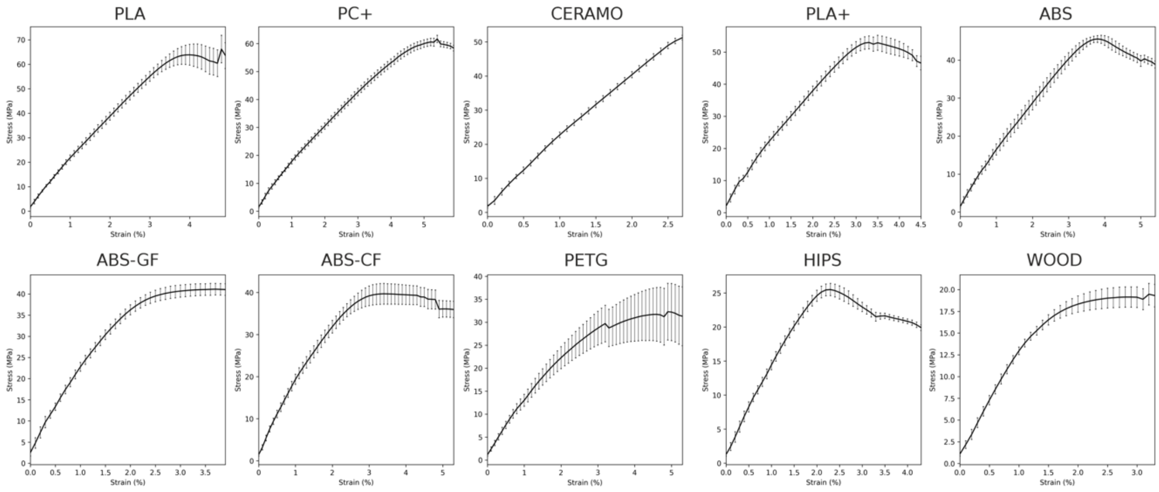

Figure S4. Aggregated stress-strain curves for the studied plastics (error bars represent standard deviations).

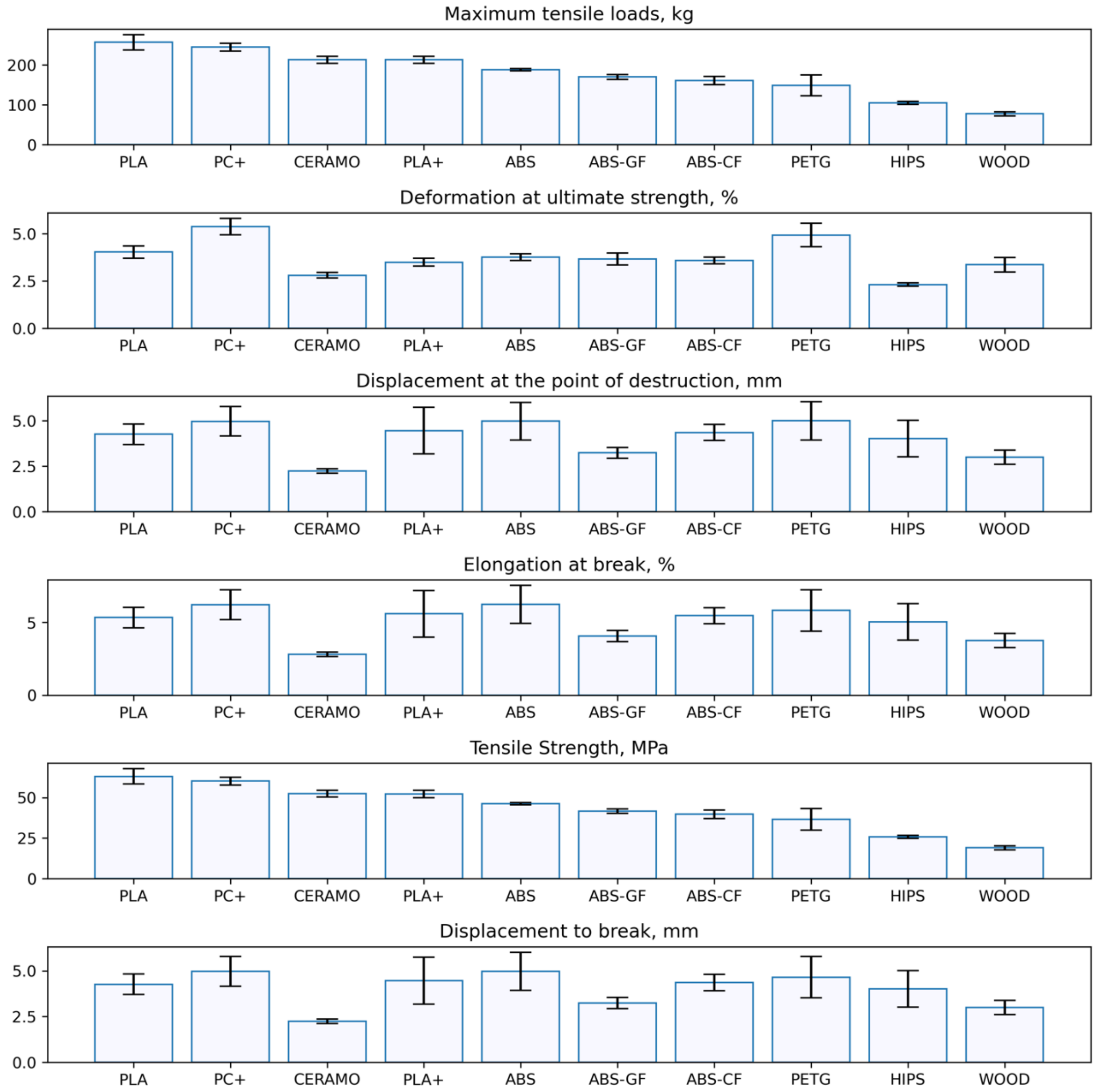

Figure S5. Mechanical properties of the plastics under study (error bars represent standard deviations).

Table S2. Comparison of the tensile strength of materials

Plastics	Tensile strength, MPa	Tensile strength from the manufacturer, MPa
PLA	63.1 ± 4.7	60.0*
PC+	60.2 ± 2.4	59.7 ± 1.8**
CERAMO	52.5 ± 2.1	58.0 – 75.8*
PLA+	52.3 ± 2.3	65.0**
ABS	46.2 ± 0.7	30.0 – 57.0*
PETG	36.6 ± 6.6	52.2**
HIPS	25.8 ± 1.0	31.0*

*information taken from the MatWeb database (http://www.matweb.com/index.aspx)
**information taken from the manufacturer's website (https://www.esun3d.net; https://raise3d.ru)

Table S3. Correlation of strength characteristics and temperature

Plastics	Maximum load, kg	Tensile Strength, MPa	Maximum heating
PLA	257 ± 19	63.1 ± 4.7	2.94
PC+	245 ± 10	60.2 ± 2.4	19.65
CERAMO	213 ± 9	52.4 ± 2.1	1.09
PLA+	213 ± 9	52.3 ± 2.3	10.24
ABS	188 ± 3	46.2 ± 0.7	4.49
ABS-GF	170 ± 6	41.7 ± 1.4	1.11
ABS-CF	161 ± 10	39.7 ± 2.6	1.61
PETG	153 ± 29	37.5 ± 7.2	11.42
HIPS	105 ± 4	25.8 ± 1.0	2.56
WOOD	78 ± 5	19.1 ± 1.3	0.80

Table S4. Strength characteristics of materials with altered temperature dynamics

Material	Layer location	Strength characteristics					
		Maximum tensile loads, kg	Deformation at ultimate strength, %	Displacement at the point of destruction, mm	Elongation at break, %	Tensile Strength, MPa	Displacement to break, mm
ABS-CF + PLA	front	160	3.29	4.66	5.82	39.2	4.66
	back	156	3.35	4.17	5.21	38.2	4.17
Tape 1 [a]	front	145	4.65	4.88	6.10	35.7	4.88
	back	141	4.60	5.03	6.29	34.8	5.03
Tape 2 [b]	front	167	4.04	4.05	5.06	41.1	4.05
	back	157	4.25	4.23	5.29	38.6	4.23
Tape 3 [c]	front	146	4.38	4.41	5.51	35.8	4.41
	back	146	4.05	5.07	6.34	35.8	5.07
Tape 4 [d]	front	151	3.18	4.77	5.96	37.1	4.77
	back	151	3.30	4.32	5.40	37.1	4.32
Tape 5 [e]	front	152	3.55	4.37	5.47	37.4	4.37
	back	146	3.54	4.49	5.61	35.9	4.49
Tape 6 [f]	front	182	4.93	5.07	6.33	44.7	5.07
	back	149	4.74	3.97	4.96	36.7	3.97
Tape 7 [g]	front	158	4.39	4.70	5.87	38.8	4.70
	back	160	3.83	4.04	5.04	39.4	4.04
Tape 8 [h]	front	150	3.30	4.29	5.36	36.9	4.29
	back	147	3.36	3.53	4.42	36.1	3.53
Tape 9 [i]	front	148	3.40	4.67	5.84	36.4	4.67
	back	140	3.88	4.84	6.05	34.4	4.84
Tape 10 [j]	front	149	3.31	4.34	5.43	36.6	4.34
	back	142	3.82	4.26	5.33	34.8	4.26
Adhesive	front	140	3.15	3.41	4.26	34.3	3.41
	back	137	3.23	3.52	4.40	33.6	3.52
Rubber	front	162	4.29	4.76	5.95	39.8	4.76
	back	165	4.45	4.65	5.81	40.5	4.65

[a] Multiuse fabric waterproof PVC tape. Arrangement of reinforcing fibers: longitudinal-transverse, adhesive layer based on synthetic rubber, thickness 0.1 mm.
[b] Filament tape made of fiberglass. Arrangement of reinforcing fibers: longitudinal, adhesive layer based on synthetic rubber, thickness 0.1 mm.
[c] Adhesive reinforced polypropylene fabric backed tape. Arrangement of reinforcing fibers: longitudinal-transverse, thickness 0.17 mm.
[d] Aluminum tape. Acrylic-based adhesive, thickness 0.05 mm.
[e] Adhesive tape made of frost-resistant acrylic foam. Unreinforced, acrylic-based adhesive layer, thickness 0.05 mm.
[f] Glass fiber reinforced polypropylene adhesive tape. Arrangement of reinforcing fibers: longitudinal-transverse, acrylic-based adhesive layer, thickness 0.194 mm.
[g] Glass fiber reinforced polypropylene adhesive tape. Arrangement of reinforcing fibers: longitudinal-transverse, acrylic-based adhesive layer, thickness 0.194 mm.
[h] Aluminum tape. Unreinforced, acrylic-based adhesive.
[i] Polyethylene stationery tape. Unreinforced, acrylic-based adhesive layer, thickness 0.035.
[j] Polypropylene adhesive tape. Unreinforced, acrylic-based adhesive layer, thickness 0.045.

Table S5. Correlation of strength characteristics and temperature-modified materials

Material	Layer location	Maximum load. kg	Tensile Strength. MPa	Maximum heating
ABS-CF + PLA	front	160	39.2	0.91
	back	156	38.2	1.01
Tape 1	front	145	35.7	2.84
	back	141	34.8	1.05
Tape 2	front	167	41.1	3.40
	back	157	38.6	1.66
Tape 3	front	146	35.8	1.32
	back	146	35.8	0.97
Tape 4	front	151	37.1	2.69
	back	151	37.1	0.84
Tape 5	front	152	37.4	3.04
	back	146	35.9	1.43
Tape 6	front	182	44.7	10.60
	back	149	36.7	1.78
Tape 7	front	158	38.8	3.93
	back	160	39.4	3.85
Tape 8	front	150	36.9	5.72
	back	147	36.1	0.86
Tape 9	front	148	36.4	1.03
	back	140	34.4	1.02
Tape 10	front	149	36.6	0.91
	back	142	34.8	0.79
Adhesive	front	140	34.3	6.76
	back	137	33.6	3.79
Rubber	front	162	39.8	0.64
	back	165	40.5	1.63

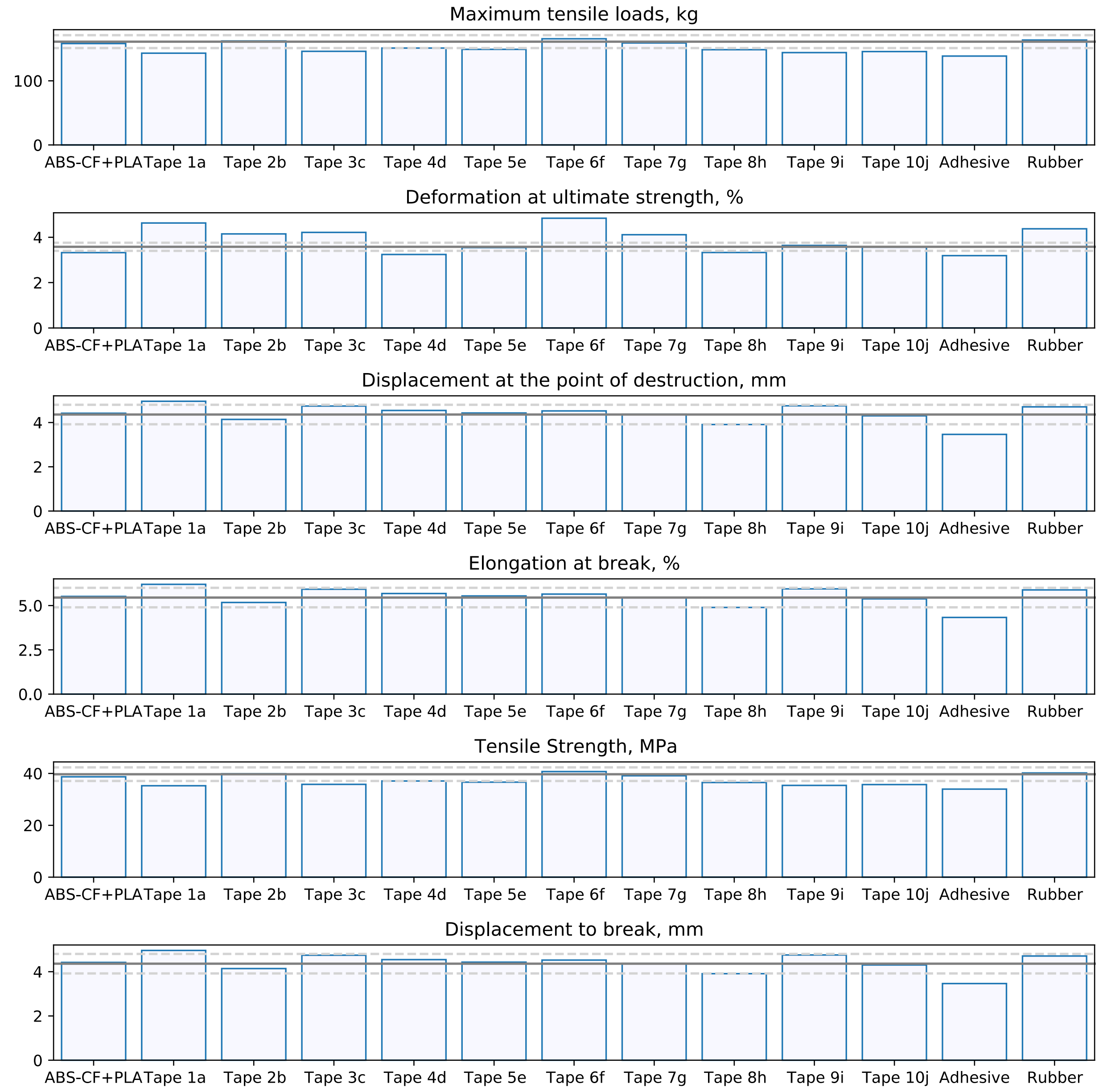

Figure S6. Impact of thermographic marker candidates on material properties (ABS-CF + PLA — result of two-layer printing, others — corresponding material is applied to ABS-CF surface). Gray lines represent values for the nonmodified ABS-CF. Dashed light gray lines represent corresponding confidence intervals for original values.

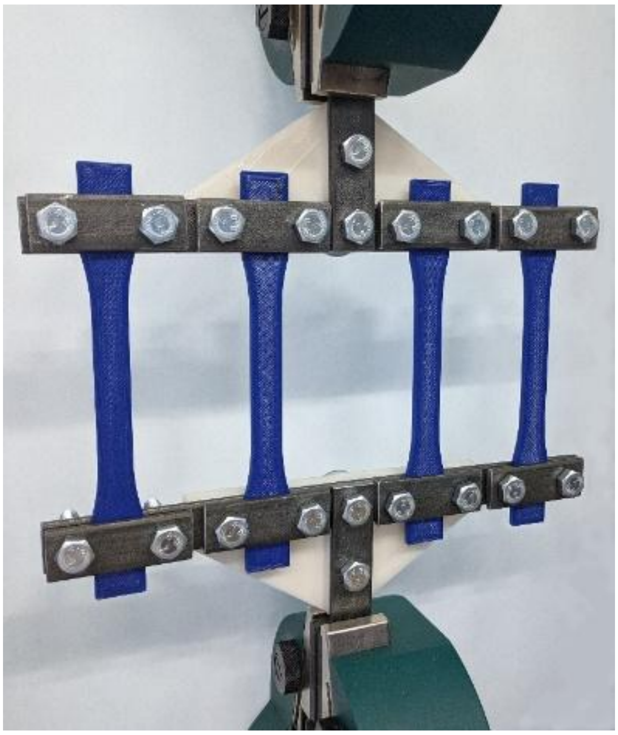

Figure S7. Hold-down built-up construction with specimens for tensile testing fixed in.

Table S6. Physical and mechanical properties of plastics tested with a built-up construction

Strength characteristics	Set 1	Set 2	Set 3	Set 4	Set 5
Maximum tensile loads, kg	512	495	487	532	499
Deformation at ultimate strength, %	8.90	9.00	8.65	10.10	9.38
Displacement at the point of destruction, mm	7.18	7.23	6.98	8.40	7.74
Elongation at break, %	8.98	9.04	8.72	10.50	9.67
Tensile Strength, MPa	126	122	120	131	123
Displacement to break, mm	7.18	7.23	6.98	8.40	7.74

Table S7. Training metrics

Architecture	Best MSE loss	Accuracy		
		5%	10%	50%
Single-frame				
256	0.0848	0.50	0.59	0.90
1024	0.0860	0.45	0.53	0.88
Multi-frame				
256	0.0659	0.65	0.74	0.94
1024	0.0756	0.65	0.73	0.94
Recurrent				
256	0.0036	0.78	0.88	0.98
256*	0.0020	0.80	0.91	**0.99**
1024	**0.0013**	0.84	0.92	**0.99**
1024*	**0.0013**	**0.89**	**0.95**	**0.99**

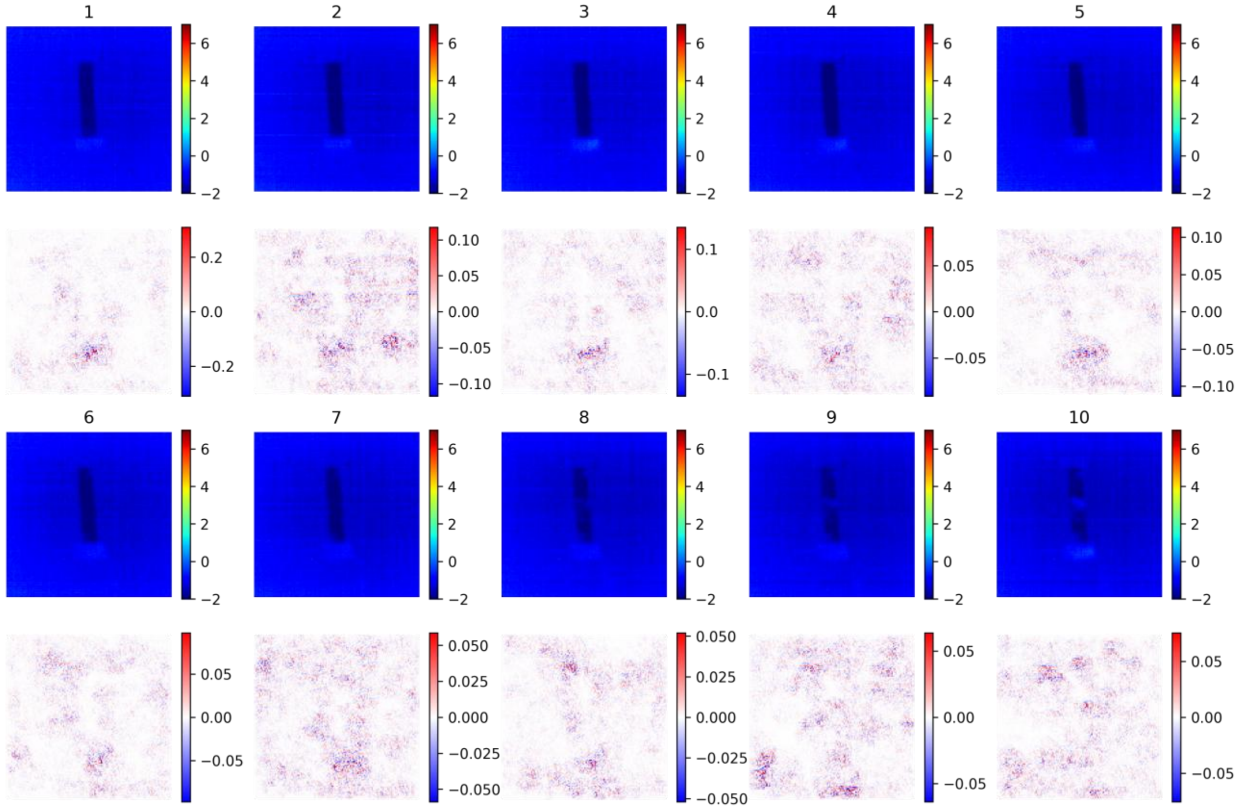

Figure S8. Gradient maps for the before-fracture prediction.

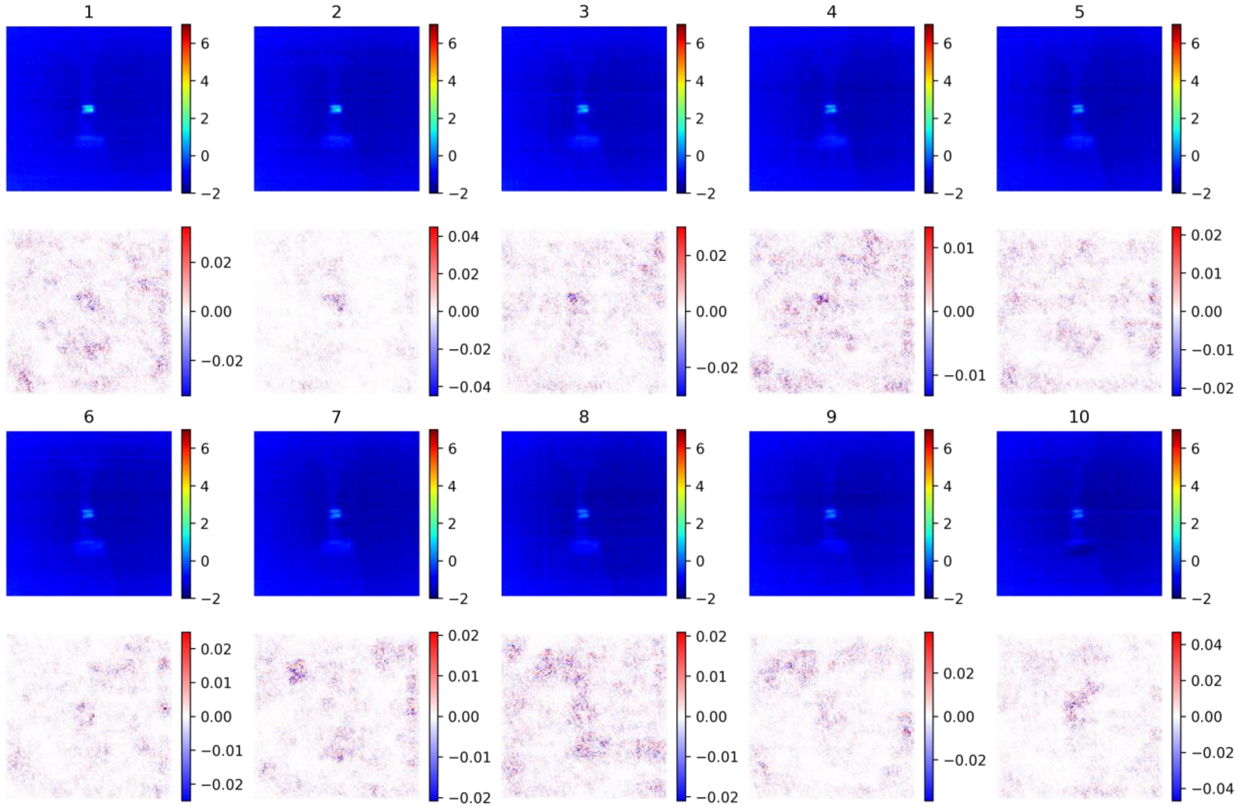

Figure S9. Gradient maps for the after-fracture prediction.
